# Fluid-Assisted Hydro-Bubble Technique With Newly Designed Cannula for Deep Anterior Lamellar Keratoplasty

**DOI:** 10.7759/cureus.93344

**Published:** 2025-09-27

**Authors:** Seiichiro Sugita, Tadasu Sugita, Iichiro Sugita, Masakazu Takayama, Hiroki Kaneko

**Affiliations:** 1 Ophthalmology, Sugita Eye Hospital, Nagoya, JPN; 2 Ophthalmology, Hamamatsu University School of Medicine, Hamamatsu, JPN

**Keywords:** big-bubble technique, cornea transplantation, deep anterior lamellar keratoplasty (dalk), hydro-bubble technique (fluid-assisted descemet’s membrane exposure), newly designed cannula

## Abstract

Purpose

This study was conducted to evaluate the efficacy and safety of the novel hydro-bubble technique (fluid-assisted Descemet’s membrane (DM) exposure) with a newly designed cannula for inducing bare DM during deep anterior lamellar keratoplasty (DALK).

Methods

This retrospective study evaluated the results of DALK in which the hydro-bubble technique (fluid-assisted DM exposure) was performed with a newly designed cannula between April 2018 and March 2020. A femtosecond laser was used to create an initial incision with minimal remaining parenchyma. Balanced salt solution (BSS) was injected into the parenchyma using the newly designed cannula to induce bare DM. The success rate of hydro-bubble formation, visual outcomes, endothelial cell density, and complications were analyzed.

Results

The study included 39 patients with corneal opacity (n=19), corneal dystrophy (n=13), or keratoconus (n=6). Hydro-bubble formation was induced in 33 (84.6%) of these patients. At one year postoperatively, visual acuity was substantially better; the mean endothelial cell density at one month postoperatively was 1847 ± 622.1 cells/mm^2^. Intraoperative microperforation occurred in six cases (15.4%), but there were no instances of macroperforation or conversion to penetrating keratoplasty. Postoperative complications included a higher incidence of double anterior chamber, which was often associated with type 2 bubbles.

Conclusion

The hydro-bubble technique (fluid-assisted DM exposure) with a newly designed cannula is a promising alternative for inducing bare DM during DALK. It achieves a hydro-bubble formation success rate similar to the rate for the conventional big-bubble technique, while allowing greater control over hydro-bubble formation size and increasing visibility during surgery. Although further validation is needed, the enhanced safety and effectiveness of the hydro-bubble technique (fluid-assisted DM exposure) can facilitate better surgical management of corneal stromal pathologies.

## Introduction

Deep anterior lamellar keratoplasty (DALK) is a therapeutic technique that is performed to replace corneal stromal pathology with healthy own endothelium [1,2]. Although various techniques have been reported, the big-bubble (BB) technique is currently the most widely used [2-5]. This technique uses a cannula or needle to inject air that forms a BB and causes Descemet’s membrane (DM) exposure [5]. The success rate of BB with a cannula alone is reported to be 54-85% [3,6-9], and it is presumably because there are a certain number of cases in which air leaks from the cannula or only the corneal parenchyma is clouded by air. For example, in cases with pre-existing rupture of DM caused by scarring after herpes keratitis, other infectious keratitis, or keratoconus with previous hydrops, air cannot successfully form a BB because it enters the anterior chamber through the fissure [4,5,9]. With the exception of cases with pre-existing rupture of DM, the success rate of BB can be increased to 90% by using preoperative anterior segment optical coherence tomography [10]. It has also been reported that the use of viscoelastic material in unsuccessful cases of BB can lead to a 100% success rate [6]. However, some improvement is required to bring the current success rate with BB for all cases closer to 100%.

While various methods for DM exposure that use air and viscoelastic materials have been evaluated, methods for DALK using fluid (hydrodelamination) have not been adequately assessed [11,12]. The hydrodelamination technique reported by Sugita and Kondo entails the application of balanced salt solution (BSS) to the cornea, which causes it to swell and become translucent, enabling the surgeons to operate more easily than with air [11]. Therefore, the hydrodelamination technique potentially enables us to reach a 100% success rate in DM exposure. However, hydrodelamination has not been widely performed due to technical difficulties; their method in the induction of bare DM. To solve this problem, we developed a newly designed hydro-bubble cannula that enables us to inject BSS as easily as the BB technique using air. Therefore, the aim of this study is to examine the clinical outcomes of DALK using our newly designed hydro-bubble cannula.

## Materials and methods

This was a retrospective, non-interventional case series conducted at Sugita Eye Hospital, Nagoya, Aichi, Japan. The study was approved by the Ethics Committee of Sugita Eye Hospital (approval number: 605) and was conducted in accordance with the tenets of the Declaration of Helsinki. The requirement for written informed consent was waived by the committee under an opt-out policy because only de-identified clinical data were analyzed.

Study population

All consecutive patients who underwent primary DALK at our center during the study period were included. Eyes undergoing DALK re-transplantation were excluded. All surgeries were performed under retrobulbar anesthesia by a single corneal surgeon (S.S.) between April 2018 and March 2020.

Surgical technique

**Figure 1:**
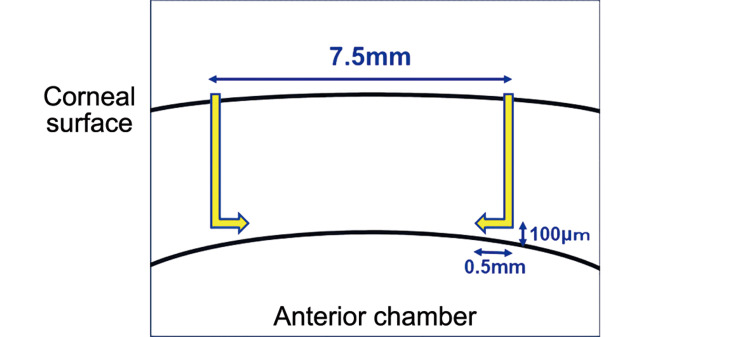
Schema showing the design of the femtosecond laser setup. The depth of the 7.5-mm wide peripheral incision is designed to leave about 100 µm, and the lamellar incision is kept at about 0.5 mm to prevent perforation in the thin central area.

The first trephination was combined with a femtosecond laser (AMO Intralase iFS; Insight Eye Equipment, Steamboat Springs, Colorado, United States) so that the thickness of the remaining parenchyma is approximately 100 μm. The laser lamellar incision from the outside to the inside was limited to approximately 0.5-1.0 mm to prevent corneal perforation during incision in cases where the pericentral cornea is thin in parts, such as keratoconus and corneal leukoplakia (Figure 1).

**Figure 2:**
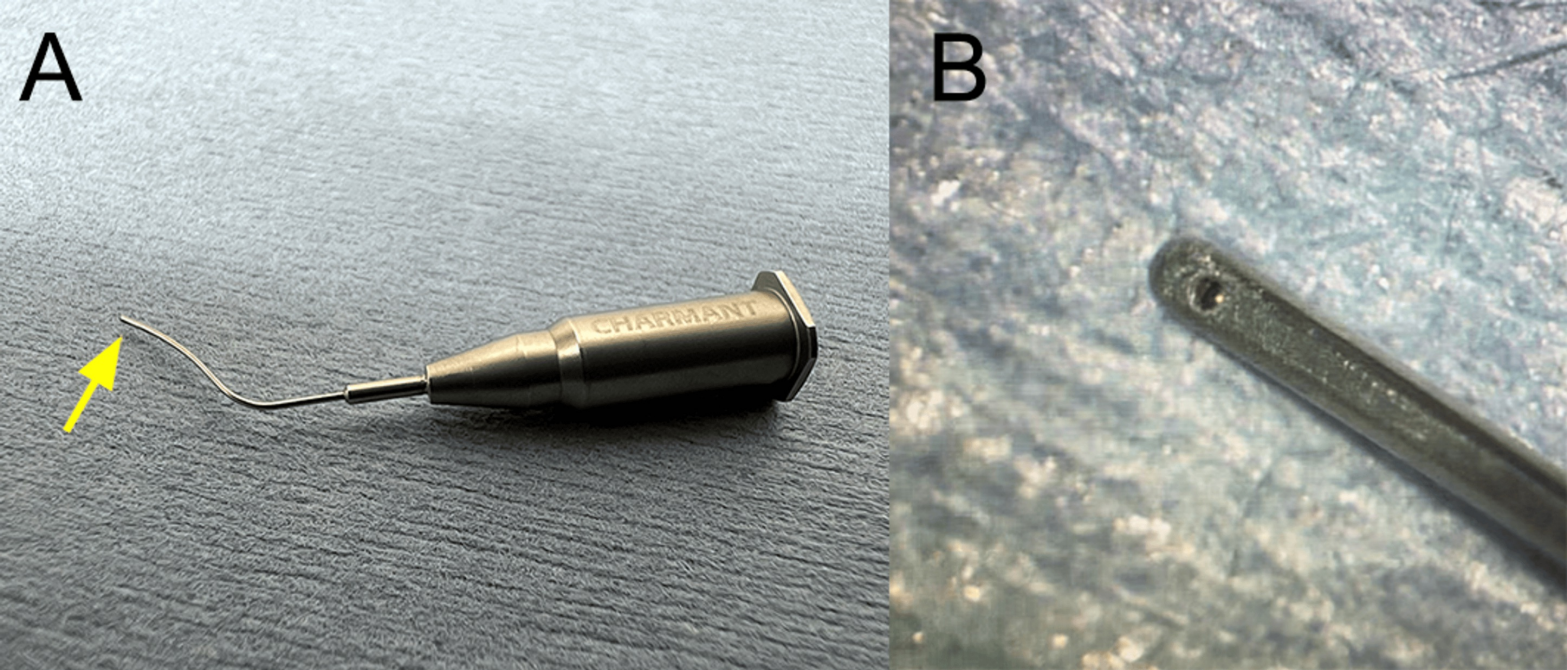
A novel hydro-bubble cannula. (A) The head of the cannula is 20% thinner and wider than a 27G needle. (B) Close-up image shows the downward hole at the tip (yellow arrow).

A golf scleral knife (MGL24; Mani Inc., Tochigi, Japan) was used to remove the first layer of the stroma. After scarring the remaining parenchyma with a golf scleral knife, a small amount of BSS was injected into the parenchyma using a Sauter needle (M-32E; Inami & Co., Ltd, Tokyo, Japan), which causes the parenchyma to swell. While injecting air in the BB technique, we injected BSS into the parenchyma for DM exposure using a newly designed cannula for the hydro-bubble technique (fluid-assisted DM exposure) (Figure 2).

**Figure 3:**
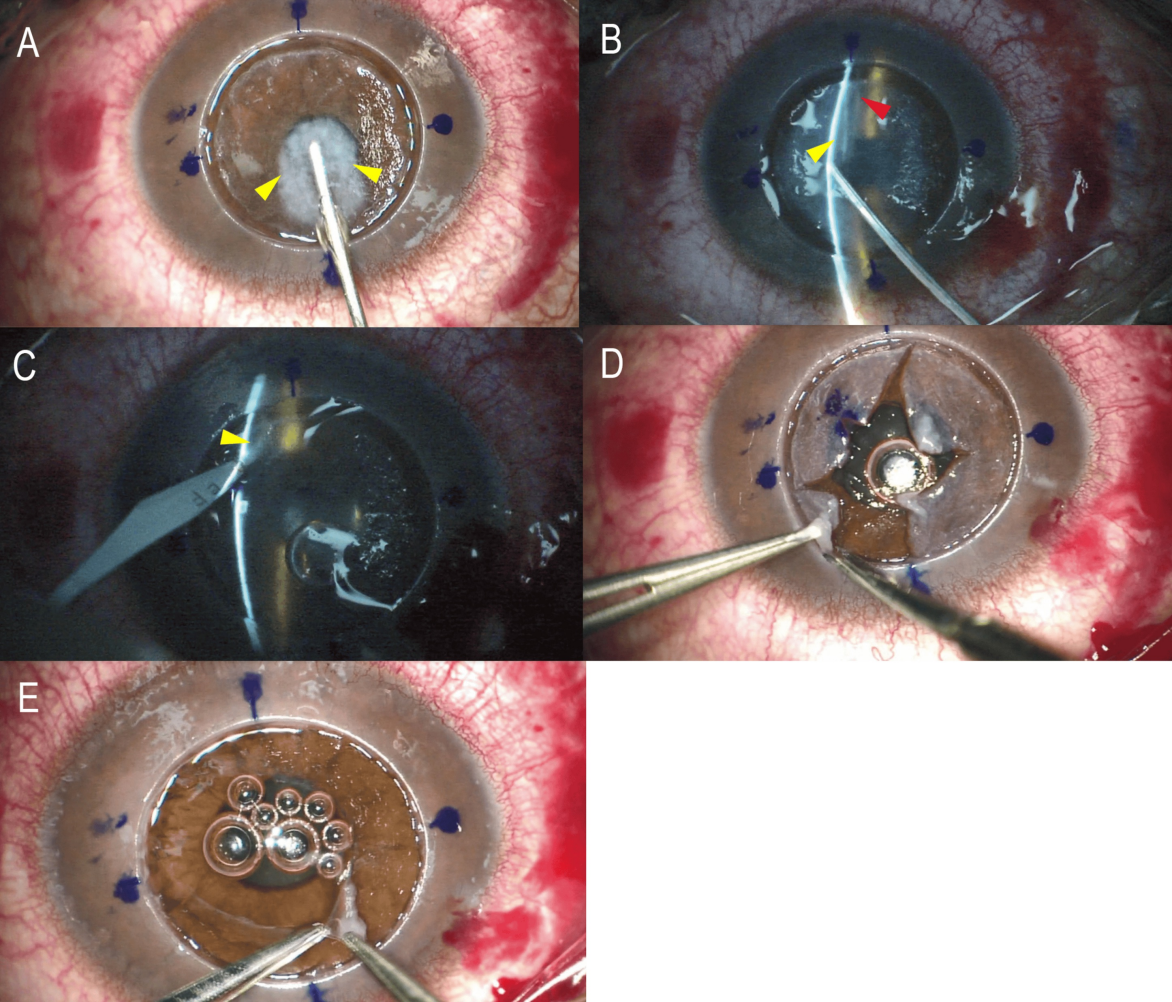
Descemet’s membrane exposure using the hydro-bubble cannula. (A) Figure showing the process of injecting BSS. When BSS is injected vigorously using the novel cannula, DM exposure was created gradually (yellow arrowheads). (B) Slit illumination under the surgical microscope showed successful fluid-assisted DM exposure using the hydro-bubble cannula. Two spaces (yellow and red arrowheads) are separated, similar to the Type 3 bubble. (C) The slit knife is used to perforate the two spaces. (D). If two spaces are separated, the residual parenchyma was carefully resected in quadrants using a spatula and scissors to enlarge the anterior space of the two spaces. (E)The exposed detached DM with a small amount of parenchyma remaining on the membrane in the periphery. DM: Descemet’s membrane; BSS: balanced salt solution

This newly designed cannula is about 20% thinner and wider across than the existing 27G cannula used for the BB technique, making it easier to enter the parenchymal interlayer, and is specifically designed for efficient BSS injection. When BSS is vigorously injected through the cannula, hydro-bubble formation gradually forms (Figure 3A).

In case hydro-bubble formation was not successful after the first injection of BSS, the cannula was re-inserted a little deeper, and BSS was injected again to achieve hydro-bubble formation. Hydro-bubble formation is clearly visible with the use of slit lamp illumination under a surgical microscope (OPMI Lumera 700 with i-OCT, Visulux; Carl Zeiss AG, Oberkochen, Baden-Württemberg, Germany) (Figure 3B), and the size of the hydro-bubble formation can be controlled by injecting BSS in cases where the area of hydro-bubble formation was smaller than that achieved by the BB technique. The slit knife (MST15, Mani Inc.) was used to perforate the stripped spaces. The anterior chamber pressure is sufficiently reduced before puncture, and the puncture site is pre-applied with a viscoelastic substance (Figure 3C). Peripheral parenchyma was manually detached while carefully swelling the stroma little by little using the hydrodelamination method using a Barraquer fine spatula with a diameter of 0.25 mm (K3-2310; Corza Ophthalmology, New Jersey, United States) and a Nagata scissors (M-50R; Inami & Co., Ltd).

If two spaces are separated, as in this case, the residual parenchyma was carefully resected in quadrants using a spatula and scissors to enlarge the anterior space of the two spaces (Figure 3D). In all cases, DALK was performed to expose DM over 5 mm of the central pupillary territory, with the goal of leaving a thin and relatively uniform parenchyma of around 60 µm in the periphery of the cornea (Figure 3E). The surgical technique is shown in Video 1.

**Video 1:** Hydro-bubble technique with newly designed cannula for deep anterior lamellar keratoplasty

Data collection

Data were collected retrospectively from the medical records and surgical logs. Collected variables included best-corrected visual acuity (logMAR), corneal topography/astigmatism, endothelial cell density (specular microscopy), anterior segment OCT images, intraoperative details (e.g., success of DM exposure, complications), and postoperative outcomes (e.g., double anterior chamber, graft clarity).

Statistical analysis

Pre- and post-operative best corrected visual acuity (BCVA) were compared using a paired t-test. Pre- and post-operative endothelial cell densities (ECD) were compared using an unpaired t-test because there were several cases in which ECD could not be measured correctly. P<0.05 was considered statistically significant.

## Results

A total of 39 patients were included: 19 corneal parenchymal opacities (48.7%), 13 corneal dystrophy (33.3%), six keratoconus (15.4%), and one corneal opacity due to Stevens-Johnson syndrome (2.6%). The mean age, primary disease, and other background characteristics of these patients are shown in Table 1**.**

**Table 1:** Preopeoperative patients’ demographic and clinical characteristics Data presented as n, except where otherwise specified *corneal opacity in the chronic stage of Stevens–Johnson syndrome logMAR: logarithm of the minimum angle of resolution; SD: standard deviation; SJS: Stevens-Johnson Syndrome; Km: mean keratometry; Kmax: maximum keratometry

Parameters	Values
Number of eyes	39
Patients’ age (years), mean± SD	68.1 ± 14
Sex (male/female)	19/20
Primary disease	
Corneal parenchymal opacity	19
- Opacity following herpetic keratitis	5
Corneal opacity due to viral infection in childhood	12
Others	2
- Corneal dystrophy	13
Lattice corneal dystrophy	12
Macular dystrophy	1
- Keratoconus	6
Km, mean (min-max)	56.3 (46.0-68.1)
Kmax, mean (min -max)	64.0 (51.2-73.7)
Classification according to the Amsler-Krumeich classification	Ⅱ：1, Ⅲ：0, Ⅳ：5
Healed hydrops	2
Others*	1
Corneal opacity in the chronic stage SJS	1
Best-corrected spectacle visual acuity (logMAR), mean ± SD	0.9 ± 0.547
Endothelial cell density, mean ± SD	2248.6 ± 538.1 cells/mm^2^

The mean best-corrected visual acuity was 0.30 ± 0.355 logMAR at 12 months after surgery, and it was significantly improved compared to that before surgery (P<0.001). The mean ECD was 1903.0 ± 631.8 cells/mm^2^ at one month postoperatively, and it was significantly reduced compared with those before surgeries (P=0.029). Mean corneal topographic astigmatism was 4.72 ± 3.21 at 12 months postoperatively. Successful hydro-bubble formation was confirmed in 33 eyes (84.6%). Analysis according to the primary disease revealed that hydro-bubble formation was successful in 16/19 eyes (84%) with parenchymal opacity, 13/13 (100%) with corneal dystrophy, and 4/6 (67%) with keratoconus.

**Figure 4:**
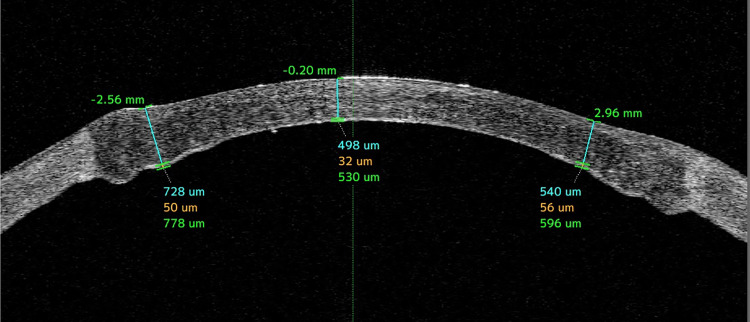
Anterior OCT photograph of the eye at two months postoperatively. Note that the residual parenchyma in the peripheral area is about 60 μm. OCT: optical coherence tomography

Hydro-bubble formation was thought to have been unsuccessful in three eyes with corneal opacity because the cannula insertion position was too shallow, and there was no apparent hydro-bubble formation. Two eyes with keratoconus, in which the procedure was unsuccessful, both had post-hydrops corneal scarring. The five cases in which hydro-bubble formation was unsuccessful were all switched to the hydrodelamination method for surgery. DM or the pre-Descemet's layer was finally exposed in 25 eyes (64.1%). Representative anterior optical coherence tomography (OCT) image two months after surgery shows that residual parenchyma in the peripheral area is about 60 μm (Figure 4).

**Figure 5:**
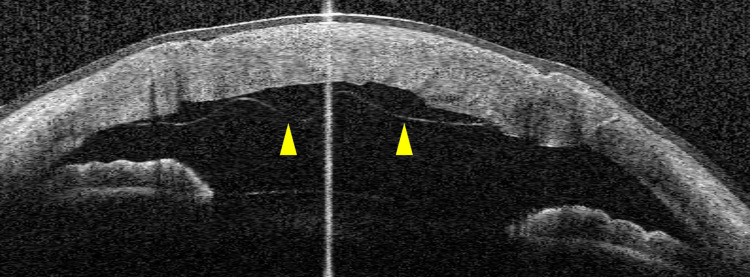
Anterior chamber OCT image from a case of double anterior chamber a few days after surgery. In this case, the second bubble (yellow arrowheads) was formed and the size of DM exposure was >8 mm. After 1–2 weeks, the double anterior chamber disappeared spontaneously. OCT: optical coherence tomography; DM: Descemet’s membrane

In some cases, two bubbles were created simultaneously. In nine eyes (23.1%), one of these bubbles was considered to be type 2. Microperforation occurred as an intraoperative complication in six eyes (15%). A double anterior chamber (Figure 5) developed as a postoperative complication in nine eyes (23%), seven of which were treated by re-injection of air. The double anterior chamber was subsequently repaired in all cases. Eight of these nine eyes had a hydro-bubble formation size greater than 8 mm. No cases were converted to penetrating keratoplasty.

## Discussion

The method of inducing DM exposure by air was first described by Archila et al. [13] and summarized as the BB technique by Anwar and Teichimann [4]. On the other hand, the hydrodelamination technique, in which the parenchyma is swollen by BSS and the DM is exposed using a fine spatula, was first reported by Sugita and Kondo [11], but was not widely used because of the difficulty in manually inducing DM exposure and a high rate of microperforation of the DM (39.2%).

In this study, we achieved hydro-bubble formation in 84.6% of cases (33/39) by using the newly designed cannula for the hydro-bubble technique (fluid-assisted DM exposure). Our success rate was comparable with or better than the rate of 54%-85% reportedly achieved by the BB technique using a cannula alone [3,6-9,14]. Analysis according to the primary disease revealed that hydro-bubble formation was successful in 16/19 eyes (84%) with corneal opacities, 13/13 (100%) with corneal dystrophy, and 4/6 (67%) with keratoconus. Excluding the cases with a history of hydrops, hydro-bubble formation was successful in all of the cases of conical keratoconus. These results suggest that our newly designed cannula gave us the same or non-inferior simplicity to the BB technique in terms of the success rate of hydro-bubble formation, and it could be beneficial, especially in cases with corneal dystrophies and dense corneal opacity.

Microperforation occurred as an intraoperative complication in six eyes (15.4%), but there were no cases of macroperforation or cases requiring conversion to penetrating keratoplasty. In previous reports, microperforation occurred at a rate of 5-19% when using the BB technique and at a rate of 0-12% when conversion to penetrating keratoplasty was needed [4,9,15]. These suggested that the rate of perforation of DM could be reduced by the hydro-bubble technique (fluid-assisted DM exposure) with a newly designed cannula [11]. The advantage of the hydro-bubble technique (fluid-assisted DM exposure) using the cannula is that it leaves a thin and relatively uniform stroma around the periphery, making it difficult for a large perforation of DM to occur peripherally. Even if microperforation does occur, the surgery can be continued and completed without problems if a thin layer of stroma can be left on top of the microperforation.

Postoperative complications included double anterior chamber in nine eyes (23%), seven of which were treated by injecting additional air into the anterior chamber. This rate indicates that the incidence of postoperative double anterior chamber might be more common in the hydro-bubble technique (fluid-assisted DM exposure) using the newly designed cannula than with the previously reported method [16]. In our study, eight of the nine eyes with double anterior chamber had type 2 bubbles. It has been reported that a double anterior chamber is more likely to occur with type 2 bubbles, which are known to occur when the Descemet stripping size is >8-9 mm [9,14,17-21]. The large number of type 2 bubbles may have resulted in the formation of numerous cases of double anterior chamber. Given that the hydro-bubble formation size can be adjusted in this procedure, the frequency of double anterior chamber might be reduced if the intention is to start creating a smaller size of hydro-bubble formation and gradually increase the size of hydro-bubble formation by injecting BSS. Similarly, when using the BB technique, the type 1 bubble is created by spreading at once [8]. However, when using the method described here, the type 1 bubble is created gradually depending on the amount of BSS injected. Therefore, it may be possible to adjust the size of the bubble with increasing experience using this method. In addition, the advantage of the hydro-bubble technique (fluid-assisted DM exposure) with a newly designed cannula is that it enables us to create a thin and relatively uniform stroma around the periphery, making large perforation of the DM in the periphery unlikely. A potential advantage of this technique is that perforation may be safer and easier to deal with than when using the BB technique.

This research had the following limitations: (i) intraoperative microscopy with slit illumination was necessary to confirm that hydro-bubble formation had been successful, (ii) inclusion of cases with a history of DM perforation may have introduced selection bias in terms of the hydro-bubble formation success rate, (iii) all the surgeries in this study were performed by single surgeon. This single-center, single-surgeon, retrospective case series with a small sample size limits generalizability. Future multi-surgeon, multi-center, prospective (ideally comparative) studies are needed to confirm reproducibility and define comparative effectiveness versus big-bubble and other DALK approaches.

## Conclusions

Our results suggest that the hydro-bubble technique (fluid-assisted DM exposure) with the newly designed cannula described here has a DM exposure success rate that is comparable with or higher than that when using earlier BB techniques and could allow DALK to be performed more safely and easily.
